# Extensive Management Promotes Plant and Microbial Nitrogen Retention in Temperate Grassland

**DOI:** 10.1371/journal.pone.0051201

**Published:** 2012-12-05

**Authors:** Franciska T. de Vries, Jaap Bloem, Helen Quirk, Carly J. Stevens, Roland Bol, Richard D. Bardgett

**Affiliations:** 1 Lancaster Environment Centre, Lancaster University, Lancaster, United Kingdom; 2 Alterra, Wageningen University and Research Centre, Wageningen, The Netherlands; 3 Institute of Bio- and Geosciences, IBG-3: Agrosphere, Forschungszentrum Jülich GmbH, Jülich, Germany; Lakehead University, Canada

## Abstract

Leaching losses of nitrogen (N) from soil and atmospheric N deposition have led to widespread changes in plant community and microbial community composition, but our knowledge of the factors that determine ecosystem N retention is limited. A common feature of extensively managed, species-rich grasslands is that they have fungal-dominated microbial communities, which might reduce soil N losses and increase ecosystem N retention, which is pivotal for pollution mitigation and sustainable food production. However, the mechanisms that underpin improved N retention in extensively managed, species-rich grasslands are unclear. We combined a landscape-scale field study and glasshouse experiment to test how grassland management affects plant and soil N retention. Specifically, we hypothesised that extensively managed, species-rich grasslands of high conservation value would have lower N loss and greater N retention than intensively managed, species-poor grasslands, and that this would be due to a greater immobilisation of N by a more fungal-dominated microbial community. In the field study, we found that extensively managed, species-rich grasslands had lower N leaching losses. Soil inorganic N availability decreased with increasing abundance of fungi relative to bacteria, although the best predictor of soil N leaching was the C/N ratio of aboveground plant biomass. In the associated glasshouse experiment we found that retention of added ^15^N was greater in extensively than in intensively managed grasslands, which was attributed to a combination of greater root uptake and microbial immobilisation of ^15^N in the former, and that microbial immobilisation increased with increasing biomass and abundance of fungi. These findings show that grassland management affects mechanisms of N retention in soil through changes in root and microbial uptake of N. Moreover, they support the notion that microbial communities might be the key to improved N retention through tightening linkages between plants and microbes and reducing N availability.

## Introduction

Humans have doubled the input of nitrogen (N) to the Earth’s land surface. The excessive use of fertiliser N has caused severe environmental problems as a result of increased gaseous N emissions from agricultural soils. This increased gaseous N loss due to denitrification contributes to climate change, as N_2_O is an approximately 300 times stronger greenhouse gas than CO_2_ [1]. Moreover, it also results in increased atmospheric N deposition and excessive N leaching from soils, which cause eutrophication of ground and surface waters, and have led to widespread changes in plant community composition and loss of plant species diversity [2]–[4]. In addition, although less extensively studied, N enrichment through atmospheric deposition or agricultural management can affect the structure and function of soil microbial communities. For example, chronic N addition has been shown to reduce soil microbial biomass and alter microbial community composition across ecosystems and biomes [5]–[7], and typically reduce the biomass of decomposer [8], [9], arbuscular mycorrhizal [10] and ectomycorrhizal fungi [11], and the abundance of fungi relative to bacteria [6]. Because soil microbes play a major role in regulating processes of N cycling [12], [13], such changes in microbial communities will have consequences for the capacity of soils to retain N, and might thus feed back to the N cycle, potentially further increasing N loss from soil. However, our knowledge of the factors that determine soil N retention, and hence the mitigation of soil N loss, is limited, despite the importance of such information for sustainable food production [13].

A long standing notion in soil microbial ecology is that ecosystems with a soil microbial community dominated by fungi have more efficient N cycling than bacterial-dominated systems [14], [15]. This concept is based on the general pattern that fungi dominate soils of undisturbed, late-successional systems of low N availability [16], and the knowledge that fungi are more efficient in their resource use than are bacteria [17], thereby slowing down rates of N cycling. Also, because of their filamentous growth form, fungi can access spatially separated C and N [18], and soils with microbial communities dominated by fungi have been shown to immobilise more added N than soils with bacterial-dominated microbial communities [19], [20]. However, results from controlled experiments assessing differences in C use efficiency and N immobilisation raise questions about the idea that fungi are more efficient in their C and N use than are bacteria [17], [21], [22], and it is possible that increased immobilisation of C and N in fungal-dominated soil microbial communities might be more a consequence of the soil conditions that they occur under in the field, rather than a physiological difference between fungi and bacteria. Land use intensification, and especially the application of fertiliser N and tillage, generally leads to a shift from fungal to bacterial dominated soil systems, although this shift is sometimes restricted to top soil [23]–[26]. In contrast, land use extensification, for instance through the cessation of fertiliser use, reductions in grazing pressure and adoption of no-tillage farming, can cause a shift from bacterial to fungal dominated systems, albeit in the long term [9], [25]–[27]. These increases in the abundance of fungi relative to bacteria due to land use extensification have been linked to more efficient N cycling and lower soil N losses [9], [20], [28], but direct support for this is lacking and the mechanisms behind the relationship between fungal abundance and soil N retention remain unclear.

Species-rich, extensively managed hay meadows are highly valued ecosystems, and the restoration of grassland biodiversity is an important aim of European Agri-Environmental schemes. A common feature of extensively managed, species-rich grasslands is that they have fungal-based food webs, which is in contrast to more intensively managed grasslands that have bacterial-based food webs [25], [29]. Grassland restoration practices such as seed addition, reduced grazing and cutting, and cessation of fertiliser application have been shown to promote the abundance of fungi relative to bacteria [30], [31]. In addition, pot experiments based on mesotrophic grasslands have shown that high plant diversity promotes soil fungal biomass with associated increases in soil N retention and reduced N loss [32], [33]. The mechanisms for these plant community composition effects on N cycling are unclear, but they likely act through linkages between plants and soil microbes [13]; plant species differentially impact on belowground microbial communities through altering the quality and quantity of organic matter entering soil via root and leaf litter and root exudates [15]. For example, it has been shown that fast-growing plant species characteristic of N-rich grasslands, which produce N rich litter and exudates, select for more bacterial-dominated microbial communities, whereas slow growing species of N-poor conditions, which produce less decomposable litter, select for fungal-dominated microbial communities, both on the level of individual plant species [34], but also on a landscape-scale [35].

Here, we investigated how changes in soil microbial communities resulting from differences in grassland management intensity affect the capacity of plants and soil to retain N. We hypothesised that extensively managed, species-rich grasslands of high conservation value would have lower N loss and greater N retention than intensively managed, species-poor grasslands, and that this would be due to a greater immobilisation of N by the biomass of a more fungal-dominated microbial community in the former. This was tested using mesotrophic grasslands of contrasting management intensity in northern England, and involved a combination of field and laboratory experiments. The field study tested whether extensively managed grasslands have lower soil N leaching losses at the landscape scale, whereas the glasshouse experiment was done to identify, through the addition of ^15^N-labelled inorganic N, the mechanisms for improved N retention in extensively managed grasslands.

## Materials and Methods

### Field Sites and Sampling

We sampled mesotrophic grasslands from eleven sites, each with an intensively managed (fertilised and grazed), species poor grassland, and adjacent traditionally managed (unfertilised, extensively grazed and cut), species-rich haymeadow of high conservation value, on the same soils (sandy silt loam) and of similar topography. The 22 mesotrophic grasslands we used were located in northern England in the region of the Yorkshire Dales (mean annual temperature 7.3°C, mean annual precipitation 1382 mm). For details of all sites used, see Table 1. Extensively managed grasslands received no inorganic fertiliser, whereas intensively managed grasslands received >100 kg N ha^−1^ y^−1^. In general, plant communities of species-rich grasslands were *Anthoxanthum odoratum*-*Geranium sylvaticum* grassland (MG3 or subcategories), and plant communities of intensively managed grasslands were *Lolium perenne*-*Cynosurus cristatus* grassland (MG6, MG7, and subcategories), according to the UK National Vegetation Classification of Rodwell [36]. In each of the 22 fields, three 1 m^2^ plots were randomly chosen within a central 25×25 m plot to avoid any edge effects. From these 1 m^2^ plots, a composite bulk soil sample was taken for assessment of moisture content, microbial biomass and community, carbon (C) and N availability, and total C and N pools. From all fields sampled, one intact soil column (18 cm height, 12 cm diameter) was taken from the centre of each plot for a field-based leaching measurement.

**Table 1:** Characteristics of the field sites used for the sampling and the glasshouse experiment; data from De Vries, et al [35].

Site	Management	Soil type	Vegetation^1^	Latitude	Longitude	Altitude (m.a.s.)	pH^2^	^15^N exp.
Askrigg Bottoms	Extensive	Sandy silt loam	MG3b	54.308398	−2.074833	307	6.0	Yes
	Intensive	Sandy silt loam	MG7c	54.308404	−2.071801	292	6.0	Yes
Waldendale	Extensive	Homose sandy silt loam	MG3b	54.206064	−2.144537	398	6.3	Yes
	Intensive	Sandy silt loam	U4b	54.244598	−1.987352	335	5.3	Yes
Yockenthwaite	Extensive	Sandy silt loam	MG3b	54.237597	−1.991726	336	5.1	Yes
	Intensive	Sandy silt loam	MG7b	54.204159	−2.1443	398	5.1	Yes
Muker	Extensive	Sandy silt loam		54.379048	−2.138109	490	5.2	Yes
	Intensive	Sandy silt loam	MG3a	54.377735	−2.138936	490	5.5	Yes
Thornton Rust	Extensive	Sandy silt loam	MG4	54.237597	−1.991726	307	5.62	
	Intensive	Sandy silt loam	MG7a	54.204159	−2.1443	307	5.7	
Church and Middlethorpe Ings	Extensive	Sandy silt loam	MG4	53.902989	−1.098635	10	6.94	
	Intensive	Sandy silt loam	OV29	53.905474	−1.093162	10	5.99	
Wheldrake and Storwood Ings	Extensive	Sandy silt loam	MG4	53.890228	−0.926907	33	6.4	
	Intensive	Clay loam	MG6a	53.880685	−0.922468	31	7.44	
Melbourne and Thornton Ings	Extensive	Sandy silt loam	MG4	53.891644	−0.847229	41	7.63	
	Intensive	Sandy loam	MG6a	53.891616	−0.847169	41	7.28	
East Cottingwith Ings	Extensive	Clay loam	MG4	53.857938	−0.944019	25	5.43	
	Intensive	Clay loam	MG7c	53.856805	−0.942937	25	6.63	
Thorganby and East Cottingwith Ings	Extensive	Clay loam	MG4	53.862402	−0.940607	28	5.16	
	Intensive	Clay loam	OV29	53.867402	−0.94702	28	5.26	
Selside	Extensive	Homose sandy silt loam	MG5b	54.168173	−2.340677	354	6.71	
	Intensive	Clay loam	MG6a	54.17053	−2.3397	334	5.1	

1 According to Rodwell [36].

2 Measured on a field level in 2005.

Permission for sampling locations was obtained from all land owners. These field studies did not involve endangered or protected species.

### Glasshouse Experiment and Sampling

Four intact soil columns (one for each treatment–control and ^15^N addition, destructive sampling after 48 hours and two months) were taken per plot from a subset of eight grasslands (four intensively and four extensively managed, Table 1) for the glasshouse experiment (with three plots per grassland this resulted in 96 columns). Columns were arranged in a randomized block design and kept in the glasshouse for one month prior to the start of the experiment, during which time they were kept at 60% water holding capacity to standardise initial soil moisture content. Two weeks before the start of the experiment, a vegetation survey was done for all columns. One week before ^15^N addition, aboveground vegetation was cut to 4 cm, and the bottom of the columns was sealed to prevent unwanted leaching. Twenty-five ml of ^15^NH_4_^15^NO_3_ solution (99.5% enriched, 24.5 mg ^15^N column^−1^, equals 30 kg ^15^N ha^−1^), and demineralised water for control treatments, was injected in the top 5 cm of each column at five, evenly spaced, locations (5 ml each). Forty-eight hours and 60 days after ^15^N vs. demineralised water injection, columns were leached in the same way as field columns, weighed, and dismantled, after which all aboveground vegetation was clipped. Columns were then divided in two: one half was used to determine root biomass, whereas the other half was used for soil and microbial analyses.

### Leachate, Soil, Vegetation, and Microbial Analyses

Columns used for the field-based leaching measurement were kept cool and immediately leached on return to the laboratory, by slowly adding 330 ml of demineralised water (equal to a 40 mm rainfall event or a heavy summer thunderstorm (MetOffice, 2012)). Columns from the glasshouse experiment were leached in the same way, 48 hours and 60 days after ^15^N addition. Leachate volumes were recorded. Vegetation was clipped and columns were divided in two for root and soil analyses. Leachates, field soil, and column soil were analysed for concentrations of inorganic N and dissolved organic carbon (DOC) and total N, as described by Gordon et al. [28]. Vegetation samples were dried at 60°C, weighed, ground, and analysed for total C and N content using an Elementar Vario EL elemental analyzer (Hanau, Germany). For the glasshouse experiment, 200 ml of leachate was freeze dried for ^15^N analysis. Microbial biomass C and N in field and column soil was determined by fumigation extraction as described by Brookes et al. [37]. For the glasshouse experiment, all N in microbial extracts was converted into ammonium by Kjeldahl digestion, which were then used to diffuse N onto an acid trap. Leachate (after freeze-drying), shoot, root, total soil, and microbial biomass ^15^N (determined by diffusing microbial derived N onto an acid trap) were analysed using a Carlo Erba NA2000 analyser (CE Instruments, Wigan, UK) and a SerCon 20–20 isotope ratio mass spectrometer (SerCon Ltd, Crewe, UK) at Rothamsted Research, North Wyke. A dried and ground grass herbage sample labelled with ^15^N (2.79 atom % ^15^N) or natural abundance wheat flour (0.368 atom % ^15^N), both calibrated against IAEA-N-1 by Iso-Analytical, Crewe, UK, were used as the references for enriched or natural abundance samples respectively. ^15^N excess atom percent values for enriched samples were calculated using mean ^15^N atom percent values of unlabelled samples [38]. These values were then used to calculate ^15^N concentrations in samples, and total amounts of ^15^N in pools were calculated using total pool sizes in columns [19], which were then scaled to kilograms per hectare using the surface area of columns.

In field samples, the biomass and structure of the soil microbial community was assessed by PLFA analysis. The fatty acids i-15∶0, a-15∶0, 15∶0, i-16∶0, 17∶0, cyclo-17∶0, 18∶1ω7 and cyclo-19∶0 were chosen to represent bacterial PLFA, 10-methyl 18∶0 and 10-methyl 16∶0 represent actinomycetes, 18∶1ω9 represents eukaryotes, and PLFA 18∶2ω6 was used as an indicator of fungal biomass [39]. The ratio of fungal to bacterial PLFA was used as an indicator of changes in the relative abundance of these two microbial groups [40]. Shifts in microbial community composition were assessed using PCA of relative abundances of all PLFAs, and Simpson’s evenness was calculated of PLFA profiles [41]. In addition, fungal and bacterial biomass were determined by epifluorescence microscopy [20]. Briefly, microscopic slides for counting fungi were stained with Differential Fluorescent Stain solution, and slides for counting bacteria were stained with the fluorescent protein dye 5-(4,6-dichlorotriazin-2-yl) aminofluorescein. Hyphal length was measured using an epifluorescence microscope at 400× magnification. Total hyphal length was calculated using the grid intersection method [42]. Fungal biomass was calculated assuming a mean hyphal diameter (width) of 2.5 mm and a specific C content of 1.3 ×10^13^ g C mm^3^ [43], [44]. Bacterial numbers, cell volumes and number of dividing cells were measured automatically with a confocal laser-scanning microscope (Leica TCS SP2) combined with image analysis software (Leica Qwin Pro), as described by [45]. Bacterial biomass (C) was estimated from the biovolume using a specific C content of 3.1 ×10^13^ g C mm^3^ [46].

### Statistical Analysis

Data were log-transformed to meet assumptions of normality. Field data were analysed using linear mixed effects models with a field level random effect to account for the nesting in fields, and true significant values were obtained by a likelihood ratio test (LRT) [47]. Apart from testing for effects of management on N and C leaching, we selected the models best explaining field N and C leaching. Parameters added to the models for nitrate availability and N leaching included management, soil properties (total C, total N, C/N ratio), plant and root properties (biomass, total N, total C, C/N ratio), and microbial properties (microbial biomass C and N, fungal PLFA, bacterial PLFA, F/B PLFA ratio, PC axis 1 (PC1) scores of all PLFAs, PC axis 2 (PC2) scores of all PLFAs, PLFA evenness). Parameters retained in the models were selected based on Akaike’s Information Criterion (AIC) and estimated significance values, after which true significance of included parameters was obtained by an LRT [48]. Glasshouse data were analysed for treatment effects using ANOVA with an error term to account for the nesting in block and field. In addition, glasshouse data were analysed using linear mixed effects models to find the parameters that best explained ^15^N leaching. Parameters added to this model were management, soil properties (total C, total N, C/N ratio), plant and root properties (biomass, total N, total C, C/N ratio), and microbial properties (microbial biomass C and N, fungal PLFA, bacterial PLFA, F/B PLFA ratio, PC1 scores, PC2 scores, PLFA evenness). Similar to the field data, parameters included were selected based on AIC and estimated significance values, after which true significance of included parameters was obtained by an LRT. R-squared values were obtained by regressing saved model predictions against observed values. For all statistical analyses, *P*-values smaller than 0.05 were considered significant. All analyses were done in R version 2.12.1 (R Development Core Team 2009).

## Results

### Field Sampling

First, we examined differences in soil and microbial properties, and C and N leaching, between the different management intensities. Intensively and extensively managed grasslands differed in some soil, microbial, and vegetation properties, but were similar for others. Inorganic N leaching was greater in intensively than in extensively managed grasslands (L-ratio = 8.51, *P* = 0.0035), whereas total N leaching, DOC leaching, and soil inorganic N did not differ between the two grassland types (Table 2). Total soil N content, and soil, root, and shoot C/N ratios did not differ between grasslands, although total soil C content and microbial biomass C and N tended to be greater in extensively than in intensively managed grasslands, albeit non significantly.

**Table 2:** Differences in C and N leaching, and soil, vegetation, and microbial properties between intensively and extensively managed grasslands.

	Intensive	Extensive	L-ratio	*P*
Inorganic Nleached (kg ha^−1^)	0.38 (0.07)	0.08 (0.02)	8.51	0.0035
Total N leached (kg ha^−1^)	1.29 (0.12)	1.00 (0.14)	1.93	0.165
DON leached (kg ha^−1^)	0.91 (0.08)	0.93 (0.14)	0.91	0.340
DOC leached (kg ha^−1^)	2.12 (0.19)	1.82 (0.16)	0.78	0.376
Soil inorganic N(mg kg^−1^)	16.9 (1.5)	16.5 (3.3)	1.58	0.209
Total soil C (mg g^−1^)	72.8 (2.5)	84.7 (5.7)	2.87	0.090
Total soil N (mg g^−1^)	6.74 (0.26)	7.59 (0.56)	1.72	0.190
Soil C/N ratio	10.8 (0.1)	11.4 (0.2)	1.72	0.189
Root C/N ratio	22.5 (1.1)	25.3 (1.3)	1.79	0.181
Shoot C/N ratio	19.7 (1.4)	22.5 (1.2)	1.22	0.269
Root biomass (kg m^−2^)	1.44 (0.15)	2.21 (0.17)	4.68	0.031
Microbial biomassC (µg g^−1^)	803 (96)	1175 (122)	2.54	0.111
Microbial biomassN (µg g^−1^)	277 (25)	387 (34)	2.82	0.093
Bacterial PLFA(nmol g^−1^)	72.2 (4.4)	92.8 (6.3)	2.77	0.096
Fungal PLFA(nmol g^−1^)	2.02 (0.22)	5.28 (0.40)	17.65	<0.0001
F/B PLFA ratio	0.027 (0.002)	0.061 (0.004)	24.93	<0.0001
PLFA evenness	0.813 (0.002)	0.815 (0.002)	0.20	0.658
Bacterial biomass(µg C g^−1^)	89.7 (5.7)	111.7 (7.5)	2.64	0.105
Fungal biomass(µg C g^−1^)	61.7 (6.9)	78.0 (7.6)	0.03	0.860
F/B biomass ratio	0.78 (0.11)	0.77 (0.08)	0.03	0.860

Values denote means (1SE), n = 66.

**Figure 1:**
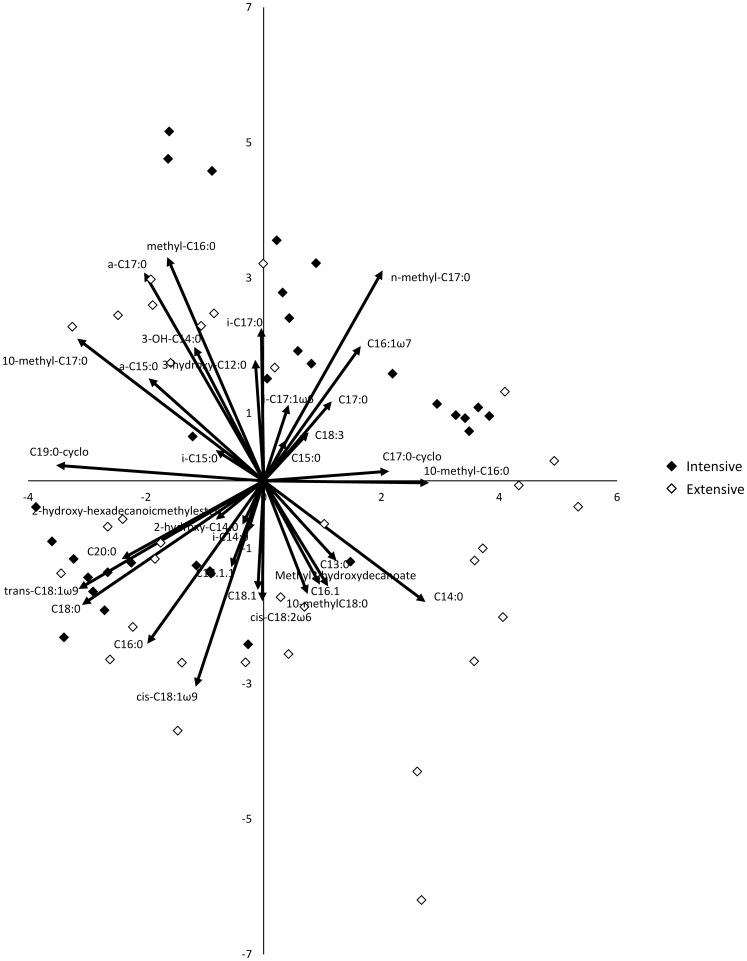
Principal components analysis (PCA) of the relative abundance of all PLFAs. PCA axis 1 explains 19.5% and PCA axis 2 explains 16% of variation in microbial community composition. Microbial community composition was not affected by grassland management.

Bacterial biomass measured as PLFA tended to be greater in extensively managed grasslands (L-ratio = 2.77, *P* = 0.096), whereas fungal PLFA was significantly (L-ratio = 17.7, *P*<0.0001) greater, and more than twice as high, in extensively than in intensively managed grasslands. As a consequence, the F/B PLFA ratio was greater in extensively than in intensively managed grasslands (L-ratio = 24.9, *P*<0.0001, Table 2). Surprisingly, microbial community composition, as assessed by PCA of all PLFAs, was not affected by management intensity, and neither was evenness of PLFA profiles (Fig. 1, Table 2). Bacterial biomass, as measured by microscopy, tended to be greater in extensively managed fields (L-ratio = 2.64, *P* = 0.105). However, fungal biomass measured by microscopy did not differ between extensive and intensive management, and as a result the F/B biomass ratio did not differ (Table 2).

**Figure 2:**
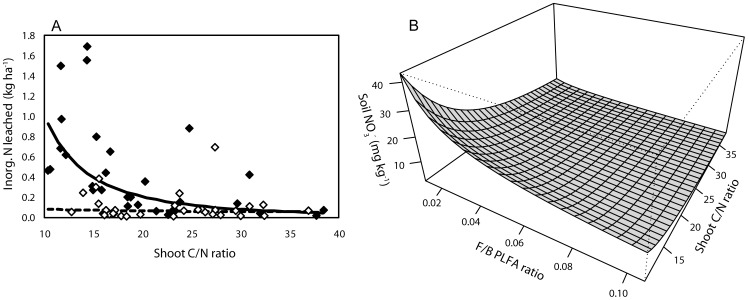
Soil inorganic N availability and inorganic N leaching from soil in the field sampling. A, Inorganic N leached in the field as explained by shoot C/N ratio in intensive (filled symbols) and extensive (open symbols) grasslands. B, Modelled relationship between soil nitrate availability, shoot C/N ratio and F/B ratio in the field. Soil C/N ratio was kept constant in the model. Soil C/N ratio *P* = 0.0025, Shoot C/N ratio *P* = 0.0004, F/B ratio *P* = 0.01. Variables were log-transformed, but axes represent true values.

Second, we used model selection (see Methods) to explore which parameters best explained C and N leaching in the field. This resulted in two satisfactory models for explaining leaching losses of N and C from soils. Inorganic N leaching was strongly explained by a model containing management intensity, (log-transformed) shoot C/N ratio, and the interaction term between the two: inorganic N leaching decreased with greater shoot C/N ratio, but only in extensively managed grasslands (Table 3, Fig. 2A). Although the models selected for DON leaching and total N leaching were significant, their explanatory power was very low (R^2^ = 0.04 and R^2^ = 0.09 respectively, Table 2). Leaching of DON was explained by a combination of management intensity, soil C/N ratio and shoot C/N ratio, and total N leached was explained by only one factor, namely soil C/N ratio. The model for DOC leaching was similar to the model explaining inorganic N leaching, with the exception that a term for microbial community was included, namely microbial biomass C (Table 3). Although inorganic N leaching was not explained by microbial community characteristics, soil NO_3_^−^ concentration was strongly explained by a combination of soil C/N ratio (Parameter Value (PV) = −2.16, *P* = 0.0025) (log-transformed), F/B PLFA ratio (PV = −0.54, *P* = 0.01), and shoot C/N ratio (PV = −0.65, *P* = 0.0004) (R^2^ = 0.48, Fig. 2B). A second model also including soil C/N ratio (PV = −2.84, P = 0.0004), shoot C/N ratio (PV = −0.73, P = 0.0002), but including PCA axis 1 scores for all PLFAs (PV = 0.05, P = 0.008), along which the relative abundance of actinomycetes increased (Fig. 1), explained a similar amount of variation (R^2^ = 0.47). In addition, inorganic N leaching was strongly explained by soil NO_3_^−^ concentration (PV = 1.06, *P* = 0.0029, R^2^ = 0.31).

**Table 3:** Selected models for inorganic N leached, DON leached, total N leached, and DOC leached in the field sampling.

	Inorganic N leached (kg ha^−1^)		DON leached (kg ha^−1^)		Total N leached (kg ha^−1^)		DOC leached (kg ha^−1^)	
	Parameter Value	*P*	Parameter Value	*P*	Parameter Value	*P*	Parameter Value	*P*
Intercept	+5.27	0.0059	−3.24	0.68	+7.55	0.037	+0.77	0.43
Management	−7.07*E	0.019	+20.2*E	0.029			−3.3*E	0.012
Soil properties			+1.42*soil C/N	0.64	−3.19*soil C/N	0.03		
			−11.6*E*soil C/N	0.0016				
Vegetation properties	−2.29*shoot C/N	0.0006	−0.13*shoot C/N	0.82			−0.73*shoot C/N	0.0087
	+1.99*E*shoot C/N	0.044	+2.4*E*shoot C/N	0.004			+1.0*E*shoot C/N	0.017
Microbial community							+0.31*microbial C	0.032
R-squared	0.46		0.04		0.09		0.28	

E = extensive management.

### Glasshouse Experiment

**Figure 3:**
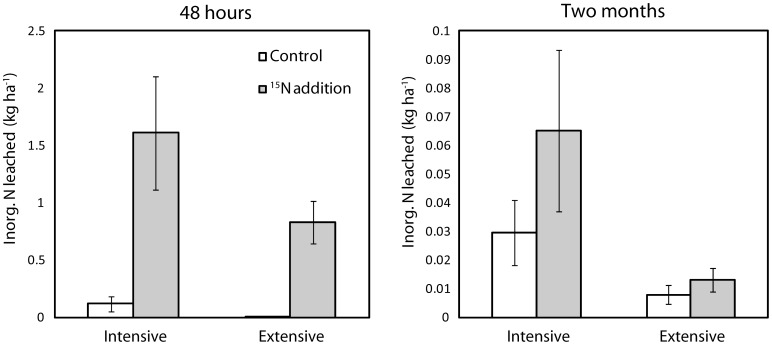
Total amounts of inorganic N leached in intensive vs. extensive soils in the glasshouse experiment, as affected by ^15^N addition and sampling date. Management F_1,86_ = 17.7, *P*<0.0001, N addition F_1,86_ = 75.7, *P*<0.0001, Sampling date F_1,86_ = 21.9, *P*<0.0001, N addition × Sampling date F_1,86_ = 45.4, *P*<0.0001. Bars represent means (n = 12) ±1SE.

**Figure 4:**
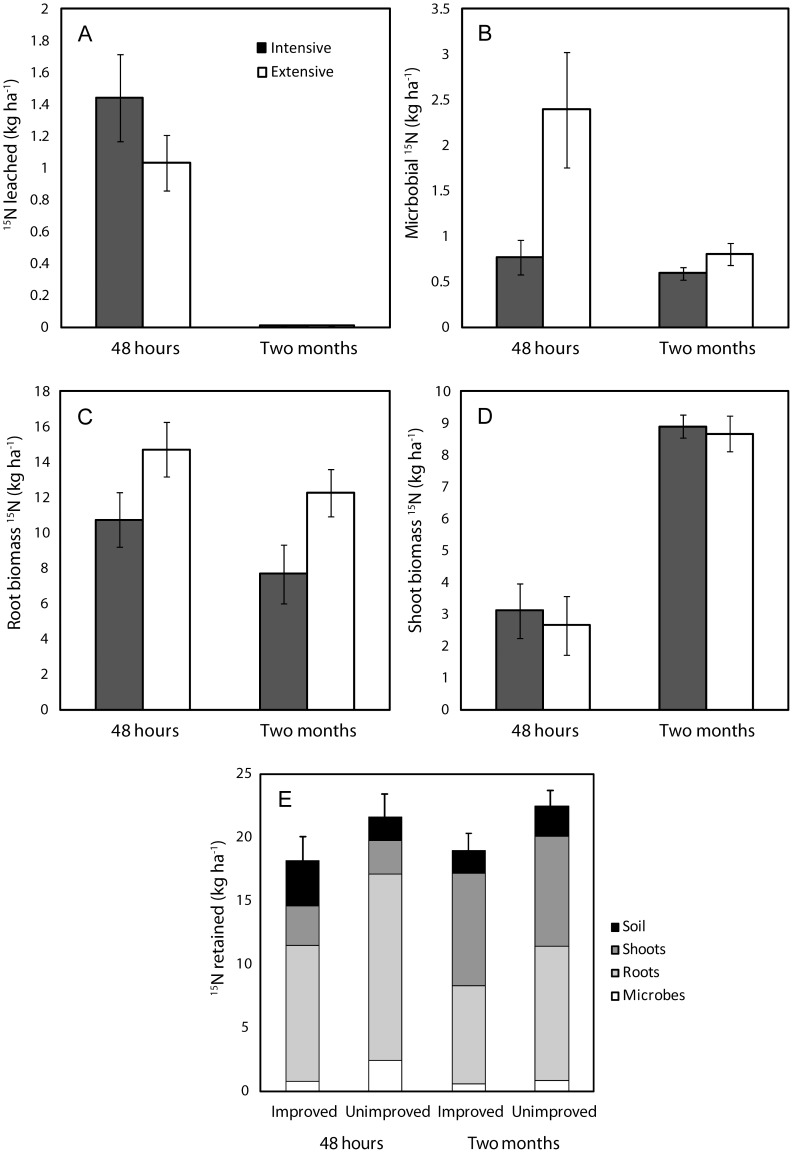
^15^N pools in intensive (black bars) vs. extensive grasslands, 48 hours and two months after ^15^N addition. A, ^15^N leached (Management F_1,42_ = 2.15, *P* = 0.15, Sampling date F_1,42_ = 58.1, *P*<0.0001, Management × Sampling date F_1,42_ = 1.61, *P* = 0.21); B, ^15^N uptake in microbial biomass (Management F_1,40_ = 7.5, *P* = 0.003, Sampling date F_1,40_ = 9.7, *P* = 0.009, Management × Sampling date F_1,40_ = 5.2, *P* = 0.03); C, ^15^N in roots (Management F_1,42_ = 6.9, *P* = 0.01, Sampling date F_1,42_ = 3.1, *P* = 0.08, Management × Sampling date F_1,42_ = 0.03, *P* = 0.85); D, ^15^N in aboveground plant biomass (Management F_1,42_ = 0.06, *P* = 0.80, Sampling date F_1,42_ = 59.6, *P*<0.0001, Management × Sampling date F_1,42_ = 0.03, *P* = 0.87). E, amount of ^15^N retained in the different pools, after 48 hours and two months (Management F_1,40_ = 5.7, *P* = 0.02, Sampling date F_1,40_ = 0.2, *P* = 0.69, Management × Sampling date F_1,40_ = 0.005, *P* = 0.94). Bars represent means (n = 12) ±1SE.

As for the field sampling, we first tested whether N leaching losses, and uptake of added ^15^N into different pools, differed between management intensities. Leaching of inorganic N in the glasshouse experiment was lower (F_1,86_ = 17.7, *P*<0.0001) from columns from extensively than from intensively managed grassland (Fig. 3). Total N leached from columns during the glasshouse experiment was strongly related to total N leaching in the field (P<0.001, R^2^ = 0.50). There was a weak trend (F_1,42_ = 2.16, *P* = 0.149) towards lower ^15^N loss from columns from extensively managed grasslands (Fig. 4A), but the total amount of added ^15^N leached did not differ between management types. However, both 48 hours and two months after addition of ^15^N, significantly (F_1,40_ = 7.5, *P* = 0.003) more added ^15^N was immobilised by the microbial biomass in extensive than intensive management (Fig. 4B). Roots took up the largest amount of ^15^N, and significantly (F_1,42_ = 6.9, *P* = 0.01) more so in columns from extensively than intensively managed grasslands; this pool only decreased slightly over time (Fig. 4C). In contrast, shoot uptake did not differ between the two management intensities and increased towards the end of the experiment (Fig. 4D). Taken together, the amount of added ^15^N retained in microbial, soil, and aboveground and belowground vegetation pools was greatest in extensively managed grasslands (F_1,40_ = 5.7, *P* = 0.02, Fig. 4E). In both systems, total retention of ^15^N did not decrease towards the end of the experiment.

**Figure 5:**
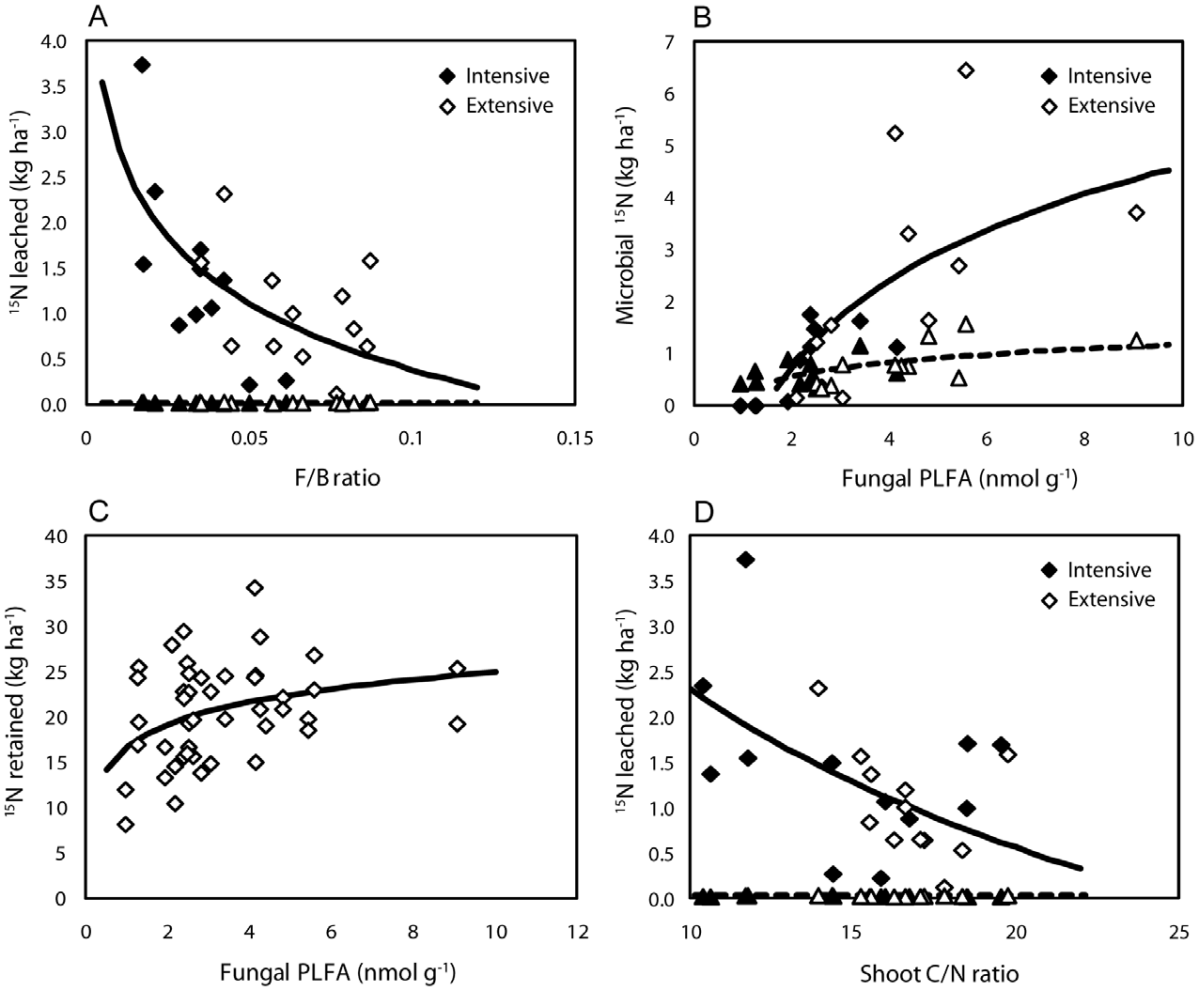
^15^N leaching and microbial ^15^N immobilisation in the glasshouse experiment. A, ^15^N leaching in the glasshouse experiment as explained by F/B ratio. Sampling date P = 0.0006, F/B ratio P = 0.0003, Sampling date × F/B ratio P<0.0001, R^2^ = 0.72. B, Microbial ^15^N uptake as explained by fungal PLFA. Sampling date P = 0.03, Fungal PLFA P<0.0001, Sampling date × Fungal PLFA P = 0.0001, R^2^ = 0.58. C, ^15^N retention in the glasshouse experiment across both sampling dates as explained by fungal PLFA (P = 0.03, R^2^ = 0.12). D, ^15^N leaching in the glasshouse experiment as explained by shoot C/N ratio. Sampling date P = 0.0006, Shoot C/N ratio P = 0.0001, Sampling date × Shoot C/N ratio P<0.0029, R^2^ = 0.67. Analyses were done on log-transformed data, but axes represent true values. Filled symbols represent improved grasslands, open symbols unimproved grasslands; diamonds represent 48-hour-sampling (except for 4C, where sampling dates are pooled), triangles two-month-sampling. Solid lines are the predicted relationship for 48-hour-sampling, dashed lines are predicted relationships for two-month-sampling.

Second, we selected the models that best explained leaching and retention of ^15^N in the soil columns of the glasshouse experiment. Leaching of ^15^N was found to decline with increasing (log-transformed) abundance of fungi relative to bacteria across all samples at both sampling dates (Sampling date *P* = 0.0006, Fungal/Bacterial (F/B) ratio *P* = 0.0003, Sampling date × F/B ratio *P*<0.0001, R^2^ = 0.72, Fig. 5A). A model including sampling date and PC2 scores explained less variation, but showed a similar pattern (Sampling date *P*<0.0001, PC2 *P* = 0.011, Sampling date × PC2 *P* = 0.06, R^2^ = 0.58); leaching of ^15^N increased with increasing PC2 scores, along which the relative abundance of fungal PLFA 18∶2ω6 decreased (Fig. 2). Immobilisation of added ^15^N into microbial biomass increased with increasing (log-transformed) fungal biomass (Sampling date P = 0.03, Fungal PLFA P<0.0001, Sampling date × Fungal PLFA P = 0.0001, R^2^ = 0.58, Fig. 5B). In addition, the retention of added ^15^N increased with greater (log-transformed) fungal biomass (Fig. 5C). Similar to the field sampling, leaching of ^15^N was explained by the (log-transformed) C/N ratio of aboveground biomass, but this model explained a smaller part of the variation in ^15^N leached than the model that included F/B ratio (R^2^ = 0.67, Fig. 5D).

Total recovery of added ^15^N in soil, vegetation and leachates was greater in columns from extensive than from intensive management (76±5% vs. 64±5%, respectively), but was not affected by sampling date. Although grassland communities in the two management types were different, the number of plant species, and the abundance of legumes, grasses, and herbs, did not differ between the columns taken from the two grassland types in the glasshouse experiment (data not shown).

## Discussion

We hypothesised that N leaching would be lower from extensively managed, species-rich grasslands than from intensively managed, species-poor grasslands, and that this would be because of a greater immobilisation of available N into microbial biomass in more fungal-dominated soils of the former. In the field experiment, extensively managed grasslands showed less inorganic N leaching than intensively managed grasslands, and the amount of inorganic N leached was best explained by a combination of grassland management and the C/N ratio of aboveground vegetation. In the glasshouse experiment, we found that extensively managed grasslands had greater retention of added ^15^N than intensive grasslands, because of a combination of greater root uptake and microbial immobilisation of ^15^N.

In the field sampling, and in accordance with our hypothesis, the fungal to bacterial PLFA ratio, and PCA axis 1 scores along which the relative abundance of actinomycetes increased, explained a significant portion of variation in soil NO_3_^−^ concentration, which is highly prone to leaching. However, inorganic N leaching was best explained by the C/N ratio of aboveground plant biomass, but only in intensively managed grasslands, which were more variable in their N leaching (Table 1, Fig. 1A). The C/N ratio of aboveground plant biomass most likely reflects differences in management within the improved grasslands, rather than differences in plant community composition, which was consistent within grassland type. There is growing evidence that plant traits, such as leaf N content, exert a strong control on belowground processes through altering the quality and quantity of organic matter entering soil [49], [50]. For instance, slow-growing plants with low leaf N content that are adapted to low-fertility conditions have been shown to decrease rates of nitrification [51] and N mineralisation [34], and to select for a more fungal-dominated microbial community [34], [35], which should decrease rates of N cycling even more [15]. Our results, therefore, point to an indirect link between plant traits, in this case leaf C/N ratio, and processes that govern N availability and leaching from soil at the field-scale. Although leaching of DOC did not differ between the two grassland types, it decreased with increasing C/N ratio of aboveground biomass similar to inorganic N leaching, which further points to the importance of plant traits for processes of C cycling. Moreover, DOC leaching increased with greater microbial biomass C, which is consistent with the notion that the microbial biomass stimulates decomposition, and thus the availability of DOC in soil, or that a C-rich soil sustains a greater microbial biomass [52], [53].

Surprisingly, although fungal PLFA and the F/B PLFA ratio were significantly greater in extensively managed than intensive grasslands, as has been shown previously [25], [29], fungal biomass and the F/B ratio, measured by microscopy, were not (Table 1). In contrast, bacterial PLFA and bacterial biomass, as measured by microscopy, both tended to be greater in extensive grasslands, and by the same magnitude (25%). An explanation for the discrepancy between fungal PLFA and microscopic counts of fungal hyphae could be that a large part of hyphae visible through a microscope might be inactive or dead [54], although the assumption that PLFAs degrade more rapidly than cell walls, and thus represent active biomass more accurately than microscopy, has been challenged [55]. Although all of our samples were treated and stored in a similar way, storage might have resulted in differences in decay of fungal hyphae and PLFAs, although this is hard to judge given that very little is known about the impacts of pre-treatment of soil samples on these methods [55], [56]. Furthermore, a general problem with PLFAs is that species composition within groups cannot be detected (for example within decomposer fungi, which are all represented by PLFA 18∶2ω6), while different species within a group might differ in their PLFA content [56], and fungal communities are likely to be impacted by grasslands management [57]. Another possibility is that fungi in extensive grassland had thicker hyphae, and thus greater membrane surface and PLFA; however, then also greater microbial biomass C would have been found. Furthermore, the PLFA 18∶2ω6 only includes decomposer fungi, while the microscopic measure also includes mycorrhiza. Although not measured here, arbuscular mycorrhizal fungi can be measured by quantifying the PLFA 16∶1ω5, although this PLFA also occurs in Gram-negative bacteria [58]. Thus, a combination of different methods is needed for a complete picture.

In the glasshouse experiment, and in support of our hypothesis, significantly more added ^15^N was immobilised into microbial biomass in extensively managed than intensive grassland soil, and ^15^N immobilisation into microbial biomass increased with increasing fungal biomass (measured as PLFA). In addition, leaching of ^15^N declined with increasing abundance of fungi relative to bacteria (F/B ratio, measured as PLFA). Although it has been suggested that fungi would immobilise available N more efficiently than bacteria, it is not possible to distinguish between ^15^N immobilised by bacteria and fungi. Therefore, although in the current study we cannot elucidate the exact mechanism, our results suggest that a greater fungal abundance is linked to increased soil N retention, a key ecosystem service in grassland. Greater microbial immobilisation of added N in extensively compared to intensively managed grasslands [19], and in fungal-dominated microbial communities compared to bacterial-dominated microbial communities [20], has been shown previously; but here we provide the first evidence that this greater microbial immobilisation of N is linked to smaller N leaching losses across a range of grassland sites.

The amount of ^15^N immobilised by microbes in extensively managed grassland soil was twice as high as the amount leached after 48 hours, which shows that microbes can be a significant short-term N sink in grassland (Bardgett et al. 2003). Roots took up the largest amount of added ^15^N, however, and significantly more so in columns from extensively managed than intensive grasslands; this pool only decreased slightly over time. Root biomass did not differ between the two grassland types in the glasshouse experiment (whereas it did in the field sampling). Therefore, arbuscular mycorrhizal fungi, which were not measured in this study, may have contributed to greater root N uptake, and also to greater N immobilisation in microbial biomass, in the extensively managed grassland. Indeed, it is known that arbuscular mycorrhizal fungi are adversely affected by intensive grassland management, including liming and fertilisation [59], and they have been shown to reduce N leaching, albeit under highly artificial conditions, and have been suggested to significantly contribute to ecosystem N retention [13]. However, as far as we are aware, there is no experimental evidence that AMF reduce N leaching under field conditions, so more work is needed to quantify their role in N uptake and recycling in grasslands [13], [29], [60], [61].

The retention of ^15^N was significantly greater in extensively managed grasslands than in intensive grasslands. Importantly, in both systems, the total retention of ^15^N did not decrease towards the end of the experiment, which suggests that the immediate N uptake in the different pools determines longer-term ecosystem N retention in mesotrophic grasslands. This is in sharp contrast with earlier results from a forest ecosystem [62], where the added N retained in soil pools after 16 weeks was only a quarter of the amount retained immediately after addition, although here aboveground N uptake was not measured. Similarly, in a study comparing N retention in urban lawns and forest, the amount of added N retained in the system after 70 days was significantly lower than the retention after one day [63]. In our experiment, ^15^N in aboveground plant biomass showed a three-fold increase during the two months of our experiment, indicating a transfer of retained ^15^N from belowground to aboveground pools. Differences in soil N retention and recovery of added ^15^N can also be a consequence of differences in gaseous N losses, which can make up a substantial amount of total N lost from soil. Our results of greater recovery of added ^15^N in extensively managed grassland soil are in line with previous findings of smaller recovery of ^15^N and greater N loss through denitrification in soils with bacterial-dominated microbial communities [20].

In conclusion, the results from our field sampling show that extensively managed, species-rich grasslands of high conservation value have lower leaching of inorganic N than agriculturally improved, species poor grasslands. Our linked glasshouse experiment showed that both roots and microbes form a stronger sink for added N in extensively managed grasslands, and that the strength of the microbial sink is related to a greater abundance of decomposer fungi relative to bacteria. This greater root and microbial uptake of N contributes to smaller N leaching losses and greater soil N retention in extensively managed grasslands. Our results advance understanding of the mechanisms of N retention in terrestrial ecosystems and how the capacity to retain N is affected by grassland management. Moreover, they support the notion that microbial communities might be the key to improved N retention through tightening linkages between plants and microbes and reducing N availability [13]. However, more detailed experiments are needed to elucidate the role of arbuscular mycorrhizal and decomposer fungi, and specific bacterial groups, in controlling N cycling processes. Pressures on land for production of food, feed and biofuel are increasing, and this has led to an urgent need to make managed systems more sustainable. Here we show that extensification of grassland management has the potential to optimize the delivery of ecosystem services like N retention, which is of central importance to sustainable food production [64], [65] and pollution mitigation [2], [3].
